# “The system always undermined what I was trying to do as an individual”: identifying opportunities to improve the delivery of opioid use services for youth from the perspective of service providers in four communities across British Columbia, Canada

**DOI:** 10.1186/s13722-022-00359-6

**Published:** 2023-01-02

**Authors:** Kirsten Marchand, Roxanne Turuba, Christina Katan, Oonagh Fogarty, Jill Fairbank, Corinne Tallon, Steve Mathias, Skye Barbic

**Affiliations:** 1 Foundry, 915-1045 Howe St, Vancouver, BC V6Z 2A9 Canada; 2 Faculty of Medicine, University of British Columbia, 317-2194 Health Sciences Mall, Vancouver, BC V6T 1Z3 Canada; 3 Department of Occupational Science and Occupational Therapy, University of British Columbia, 317-2194 Health Sciences Mall, Vancouver, BC V6T 1Z3 Canada; 4 Centre for Health Evaluation Outcome Sciences, 588-1081 Burrard St, Vancouver, BC V6Z 1Y6 Canada; 5 Canadian Centre on Substance Use and Addiction, 500-75 Albert St, Ottawa, ON K1P 5E7 Canada; 6 St. Paul’s Hospital, Providence Health Care, 1081 Burrard St, Vancouver, BC V6Z 1Y6 Canada; 7 Providence Research, 1190 Hornby St, Vancouver, BC V6Z 1Y6 Canada

**Keywords:** Youth, Opioid use disorder, Opioid use, Service providers, Human-centered co-design, Community-based participatory research

## Abstract

**Background:**

Substance use among youth is a longstanding global health concern that has dramatically risen in the era of highly toxic and unregulated drugs, including opioids. It is crucial to ensure that youth using unregulated opioids have access to evidence-based interventions, and yet, youth encounter critical gaps in the quality of such interventions. This study aims to address these gaps by identifying opportunities to improve the quality of opioid use services from the perspective of service providers, a perspective that has received scant attention.

**Methods:**

This community-based participatory study was conducted in four communities in British Columbia (Canada), a province that declared a public health overdose emergency in 2016. Human-centered co-design workshops were held to understand service providers’ (n = 41) experiences, needs, and ideas for improving the quality of youth opioid use services/treatments in their community. Multi-site qualitative analysis was used to develop overarching experiences and needs themes that were further contextualized in each local community. A blended deductive and inductive thematic analysis was used to analyze the ideas data.

**Results:**

Three overarching themes were identified, reflecting service providers’ goals to respond to youth in a timely and developmentally appropriate manner. However, this was significantly limited by organizational and systems-level barriers, revealing service providers’ priorities for intra- and inter-organizational support and collaboration and systems-level innovation. Across communities, service providers identified 209 individual ideas to address these prioritized needs and improve the quality of youth opioid use services/treatments.

**Conclusion:**

These themes demonstrate a multi-level tension between macro-level systems and the meso-level organization of youth opioid use services, which undermine the quality of individual-level care service providers can deliver. These findings underscore the need for a coordinated multi-level response, such as developing youth-specific standards (macro-level), increasing inter-organizational activities and collaboration (meso-level), and creating programs that are specific to youths’ needs (micro-level).

## Introduction

Substance use among adolescents and young adults, collectively referred to as youth (ages 12–24), is a longstanding global health issue for families/caregivers, communities, and policy makers. Substance use typically begins and peaks during this period and can impede cognitive, emotional, and psychosocial development, potentially leading to further adverse outcomes (e.g., riskier substance use patterns, health burdens) [1–4]. Global concerns for youth substance use have evolved over time as they are heavily influenced by local drug markets and policies that affect the availability of substances and their associated risks [2, 5]. These concerns have risen in the era of highly toxic and unregulated opioids (e.g., fentanyl, carfentanil), which have dramatically increased rates of preventable drug toxicity deaths among youth in North America [6, 7]. Thus, it is crucial to ensure that youth using unregulated opioids have access to evidence-based substance use services/treatments to alleviate these risks and improve youths’ well-being.

To that end, evidence-based guidelines recommend that youth using opioids and other substances have access to the same standards and treatments that are available for adults [8–11]. This includes psychosocial and pharmacological treatment, harm reduction, and long-term supports that are tailored to youths’ needs and preferences [10, 11]. While youth-focused research on opioid use treatment is scant and emerging [12], the available evidence has consistently shown worrisome disparities in youths’ engagement with such treatments [13–20]. For instance, a recent systematic review found that youth were less likely to access medications for opioid use disorder (MOUD; e.g., methadone, buprenorphine) than other age groups, with MOUD access further differentiated by broader structural inequities, such as race and socioeconomic status [15]. This gap appears to persist even after youth experience non-fatal drug toxicity events, as demonstrated by a recent population-based study where close to 70% of youth received no treatment within 30 days of such an event [17].

These disparities may be partly explained by the limited availability of youth-oriented services/treatments [13, 21]. For example, youth accessing adult-oriented harm reduction services have described experiencing stigma from both adults and service providers, which deters service engagement [21]. Another potential source for these disparities is the incongruence between youths’ treatment goals and preferences and the procedures and policies through which treatments are delivered. Studies have shown that youth have diverse goals when accessing MOUD, such as reducing opioid use, MOUD tapering, gaining employment, and improving mental health, that are not always compatible with current practices or standards (e.g., focus on abstinence and withdrawal symptoms, long-term retention) [13, 18, 22–24].

Recently, these gaps have motivated several calls to action to improve the quality of opioid use services/treatments for youth [13, 23, 25–27]. Examples include expansion of youth-led and youth-dedicated programs and services, non-stigmatizing and person-first language when delivering services, and youths’ self-determination in treatment decision-making [13, 23, 27]. These calls to action have significant implications for service providers/clinicians whose primary role is to implement and deliver care according to these recommendations. Despite this role and the expertise that service providers bring to understanding these issues, few in-depth studies have explored their experiences, needs, or ideas for improving the quality of youth opioid use services.

This qualitative study addresses this gap by adding service providers’ perspectives of the critical needs and solutions for improving the quality of opioid use services/treatments for youth. Specifically, this study asks, *What are service providers’ perspectives of the opportunities for improving the delivery of opioid use services and treatments for youth?* Findings from this study can be used to further understand, complement, and enact the growing calls to action.

## Methods

### Design and sample

Improving Treatment Together (ITT) is a multi-phase project that aims to co-design health service innovations to improve youth opioid use services through youth, caregiver, and service provider engagement using community-based participatory research (CBPR) methods. The study design has been extensively described elsewhere [23, 25, 28]. Briefly, the project commenced in 2018 through a partnership between a national (Canada) and provincial organization (Foundry, British Columbia; BC), who then identified four community-based partners that provide mental health and substance use services to youth. Phase 1 of the project (described herein) involved four separate community-based workshops with service providers in BC, a province that has faced significant burdens attributed to the unregulated and toxic opioid supply. These workshops were conducted in November 2019 and February 2020 (pre-COVID-19 pandemic).

To be eligible, participants had to self-identify as a service provider delivering services or treatments to youth ages 16–24 who use non-prescribed opioids. As youth who use opioids receive psychosocial, pharmacological, or harm reduction services in many settings (e.g., community health centers, hospitals, etc.) and from diverse professionals (e.g., counselors, peer supporters, nurses, physicians), no further criteria were specified to ensure this diversity was represented. Service providers were recruited with the support of the provincial and community-based partners, who distributed information about the study within their agencies and throughout their wider networks.

### Communities

In Canada, health care policies are developed by provincial governments (system or macro-level) and determine the delivery of opioid use treatments/services at the local community (organizational or meso-level) and individual practice setting (patient care or micro-level). To ensure diverse local perspectives were included in the project, four communities were purposefully selected based on an environmental scan performed by the national and provincial project partners. The aim was to select communities that were facing high rates of opioid-related drug toxicity events throughout the province of BC and spanning all five regional BC health authorities, including rural areas given that they often experience higher rates of overdose deaths and have less harm reduction services available [29, 30]. At the time of Phase 1, the drug poisoning death rate per 100,000 was highest in Vancouver (57.2), followed by Prince George (52.7), Kelowna (31.8), and Victoria (31.2) [31]. Thus, these four main service hubs were chosen and invited to partner in the study: Kelowna (Interior Health), Prince George (Northern Health), Victoria (Island Health), and metro-Vancouver (Vancouver Coastal Health and Fraser Health).

Vancouver is unique as it is the largest urban centre in the province and has a high concentration of health services, substance use treatment centres, and harm reduction programs within its downtown. The average income varies significantly across Vancouver’s neighborhoods, with particularly low rates in the Downtown East Side, a neighborhood that is recognized as having one of the largest urban drug scenes in North America, with a high prevalence of drug use, crime, homelessness/housing insecurity, and infectious diseases [32]. Unlike Vancouver, the other three communities serve as health service hubs for many smaller neighboring communities in a large geographical region, but hold fewer substance use services and programs. All communities have a low prevalence of racialized communities, except for Prince George, which serves a larger population of Indigenous Peoples.

### Procedures and data collection

Ethics approval was obtained from the University of British Columbia/Providence Health Care research ethics board (H19-02077) prior to any data collection taking place. Upon arrival to the workshop, participants provided fully informed consent and completed a brief socio-demographic questionnaire. The full-day workshops (one per community) were structured around the core elements of human-centered co-design [33, 34]. During the first half, participants convened into small discussion groups (4–6 participants/group) to reflect and discuss their experiences delivering services to youth who use opioids (Empathy Session). Based on their shared experiences, service providers identified and discussed the root problems to be addressed (Needs Session). During the second half of the workshop, participants brainstormed potential solutions to the prioritized needs (Ideation Session). Small group discussions were facilitated by members of the research team (authors CK, CT, OF) trained in human-centered co-design and focus group methods. Each session’s small group discussion ranged from 30 to 90 min and was audio-recorded and transcribed verbatim. Flipcharts and white boards also supported individual reflection and group discussions.

### Analysis

The primary data source for the analysis includes the small group discussion transcripts (n = 10). Images of the flipcharts and white boards (n = 17) were also used to support theme development. The analysis was led by first author (KM), who has extensive experience with qualitative data analysis in health services and substance use research. The analytic approach was decided upon careful reading of the data and consultations with the data collection team. Throughout the analysis, a reflexive journal was kept to document reflections, insights, and methodological decisions.

For the Empathy and Needs session data, a multi-site qualitative analysis [35] was conducted with the goal of identifying overarching themes for all sites, while remaining attentive to local site-specific patterns. This approach involves a ‘within-between-within’ method [35]. For the first within-site analysis, an in-depth inductive thematic analysis was used to develop site-specific themes of participants’ experiences and needs for improving youth opioid use services [36]. During this analysis, memos were used to reflect on potential similarities and differences between sites. For the between-site analysis, the within-site themes and memos were studied and discussed with the team to identify points of intersection and divergence between the four communities. This led to the development of three semantic overarching themes. In the second within-site analysis, each community’s transcripts were re-analyzed according to the overarching themes to further explore each theme’s characteristics within the four communities.

Data from the Ideation session emerged directly from the Needs session and involved less in-depth discussion. Thus, a blended deductive and inductive approach was used. After coding each individual idea from across the four communities, the overarching themes were used to deductively sort the ideas codes. Then, within each overarching theme, the ideas were thematically analyzed using an inductive approach to identify sub-themes of solutions within each overarching theme. Theme identification, definition, and interpretation were reviewed and discussed with two service providers, one of whom was present in the workshops and another who has provincial expertise in the delivery of opioid use services/treatments for youth.

## Results

A total of 41 service providers participated in the four workshops, their characteristics are shown in Table 1. Briefly, participants represented a diversity of health professions, primarily counseling/social work (46%), nursing (16%), and peer support/navigation (16%) and worked in integrated youth services (46%), community health (36%), or outreach settings (33%). The most frequent types of substance use services/treatments provided to youth included psychotherapeutic interventions (74%, e.g., motivational enhancement therapy), harm reduction (74%), and screening or early intervention (61%).

**Table 1:** Characteristics of service provider participants in the four community-based workshops* (n = 41)

Workshop characteristics^a^	N (%)/median (Q1, Q3)
Total number of participants in each community	41 (100)
Victoria	13 (32)
Vancouver	12 (29)
Prince george	12 (29)
Kelowna	4 (10)
Number of small discussion groups in each community^a^	10 (100)
Victoria	3 (30)
Vancouver	3 (30)
Prince George	3 (30)
Kelowna	1 (10)
Participant socio-demographic and occupation characteristics (N = 39)^b^	
Gender^c^	
Woman	32 (82)
Man	7 (18)
Ethnicity^d^	
White/caucasian	31 (80)
First Nations, inuit, métis	6 (15)
Other, including East Asian, South Asian, Hispanic	4 (10)
Median age (Q1, Q3)	41 (34, 48)
Occupation^e^	
Counselor or social worker	17 (46)
Registered nurse or nurse practitioner	6 (16)
Physician	3 (8)
Peer or family peer support and navigation	6 (16)
Youth outreach educator	1 (3)
Program manager or program administration	4 (11)
Median years in occupation (Q1, Q3)	7.5 (3, 15)
Median years working with youth	10 (4.5, 17)
Primary practice setting^d^	
Hospital-based setting (inpatient, outpatient, emergency department)	10 (26)
Outreach setting	13 (33)
General community health centre	14 (36)
Integrated youth services centre	18 (46)
Private practice/office-based setting	3 (8)
School-based setting	5 (13)
Substance use services and treatments provided^d^	
Screening or early intervention	24 (61)
Brief intervention	21 (53)
Individual psychotherapeutic interventions^f^	29 (74)
Group or family based psychotherapeutic interventions^g^	22 (56)
Vocational or occupational services	8 (20)
Peer support services	4 (10)
Harm reduction services	29 (74)
Pharmacological treatment	19 (48)

*SD* standard deviation, *Q1* 25th percentile, *Q3* 75th percentile

^*^ Workshops occurred in four communities across five health regions in British Columbia: Kelowna (Interior Health), Prince George (Northern Health), Victoria (Island Health), and metro-Vancouver (Vancouver Coastal Health and Fraser Health)

^a^Participants self-selected into smaller discussion groups, ranging from 3–5 participants each

^b^The socio-demographic survey was voluntary and the response rate was 95% (39/41 completed; 2 missing, 1 in Vancouver and 1 in Prince George)

^c^Response options also included the following, but were not selected by any participants: Non-binary, Two-spirit, Trans female, Trans male, Not sure/questioning, Prefer not to answer, Other

^d^Participants could choose more than one response option, and therefore do not sum up to 100%

^e^n = 37, 2 missing responses

^f^Collapsed category includes cognitive behavioural therapy, dialectical behavioural therapy, contingency management, motivational interviewing, motivational enhancement therapy, and/or mindfulness-based relapse prevention

^g^Collapsed category includes family therapy, mutual aid groups, and/or psychoeducational groups

### Overarching empathy and needs themes

**Fig. 1:**
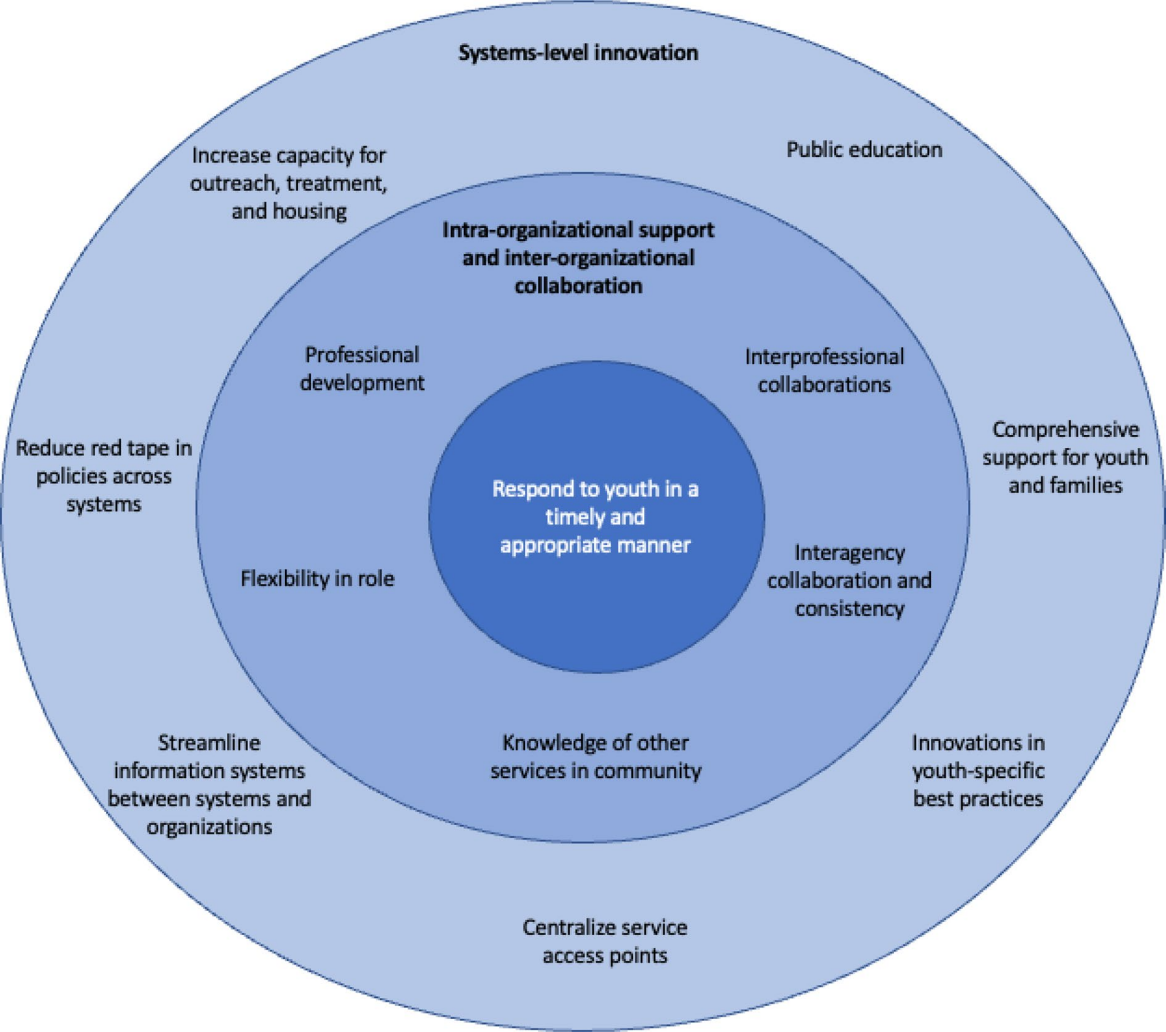
Overarching themes for improving the delivery of opioid use services/treatments for youth

The multi-site qualitative analysis led to the identification of three overarching themes (Fig. 1) that provide an in-depth understanding of service providers’ experiences delivering youth opioid use services and needs for improvement. Across the four communities, there was a strong point of connection in service providers’ primary goals to respond to youth in a timely and appropriate manner. However, this goal was met with significant intra- and inter-organizational barriers. To overcome these barriers, participants prioritized intra-organizational opportunities, such as professional development and flexibility in their roles, and stronger inter-organizational knowledge and collaboration between agencies and professionals. Ultimately, higher systems-level limitations shaped these organizational needs and service providers’ ability to meet their goals. As expressed by one participant, *“in a big system, I always felt defeated. The system always undermined what I was trying to do as an individual”* (Participant 3, small group 1, Prince George). Systems-level innovation to address these needs included increased capacity for services, innovations in youth-specific best practices, and comprehensive support for youth and families/caregivers.

### Responding to youth in a timely and appropriate manner

Across the four communities, service providers’ primary goal was to provide youth with services/treatments that best respond to *“what the youth wants and their circumstances”* (Participant 2, small group 2, Victoria). Participants in Kelowna and Vancouver specifically described working with youth who have complex needs (e.g., pain, concurrent stimulant use, mental illness, cognitive impairements, homelessness), which meant that they were trying to match services to “*the most bothersome thing for them at the time”* (Participant 3, small group 2, Vancouver) while also focusing on youths’ imminent safety due to the toxic drug supply.

Participants discussed how service environments were not tailored to youth who use drugs. This included a lack of service settings that are inclusive for diverse youth, including Indigenous youth and 2SLGBTQIA + youth, as described by service providers in Prince George and Victoria. Critical obstacles to meeting youths’ needs also included treatment policies and procedures that were not realistic for youth (e.g., the unrealistic focus on abstinence) and the limited availability of services when referrals were made or in moments when youth were open to engage. Service providers described having to “*compromise*” in relation to the guidelines and contructs of their professional practice that affected their ability to provide client-centered care. As expressed by this participant, this was difficult to navigate, particularly when youths’ safety might be at risk.

*“… how can I get creative with the situation while still providing safe care within these guidelines and this construct that I have to practice in? And how can I like think outside the box and still create a solution, but still, you know, follow the boxes and things that I need to click?…There are certain things that I need to make sure to happen and sometimes it's difficult to creatively get outside of that box and still make those things happen.”* (Participant 4, Kelowna).

Without youth-tailored environments, service providers discussed how youth may disengage from services, and thus, aimed to establish good rapport and positive relationships to encourage feelings of safety, decrease shame and stigma, and encourage youth to return. In doing so, they used several different strategies, such as offering bus tickets and food, showing unconditional support, and applying strengths-based techniques.

*“I’m trying to build a relationship so that they keep coming back. Because there's not actually a lot that like draws youth to our space, other than like needing supplies and like gear, so some of them will just come and get stuff and then like go, and so I’m trying to build a relationship, so they'll come and seek me out”.* (Participant 5, small group 2, Victoria).

To improve service availability, participants prioritized a wider range of “*youth-specific services along with a continuum*” (Participant 1, small group 3, Vancouver), which included drug checking, safe supply, harm reduction approaches within treatment centres, more MOUD options, interventions for youth using stimulants (e.g., amphetamines), detox and residential treatments, and culturally safe and relevant options that are Indigenous-led. Participants also prioritized improved treatment accessibility through reduced waiting times, longer operating hours (e.g., weekend hours), and transportation between and within communities. Lastly, providers identified the need for safer and more private service delivery environments and opportunities for *“the client [to] inform the provider, it's not like the provider's the expert”.* (Participant 3, small group 1, Prince George).

### Intra-organizational support and inter-organizational collaboration

Participants in each community described encountering organizational silos and poor communication between individual service providers. These challenges resulted in wasted time and resources, inconsistencies between providers, limited trust and confidence in service partnerships, and poor care continuity. As expressed by this participant:

*“Physicians have very different prescribing practices…I shadowed at the needle exchange for an afternoon at their OAT [opioid agonist treatment or MOUD] clinic and I found our approaches were quite wildly different, but it was good to know when you're talking to a client, like ‘this is what to expect over here, like this will meet your needs in a way we can't or *vice versa*’”.* (Participant 1, small group 2, Prince George).

Accordingly, participants identified the need for streamlined communication between different service providers and agencies who may be working with the same youth, which would improve youths’ interprofessional support system and handoffs and transitions between providers. This was also identified as a way to encourage resource sharing and fill the gaps they faced with limited organizational supervision and training in best practices.

*“If there was some sort of opportunity with interagency case planning… I’m working with a youth right now who yeah has lots of support services, like has a worker, has me, has another outreach worker, social worker, counselor and trying to get them into treatment and we're all like calling, like all four of us are e-mailing the social worker about the same thing and it's just madness. It's inefficient and it's defective”.* (Participant 2, small group 1, Victoria).

Within their own organizations, participants discussed time as a major limitation in their ability to build relationships with youth and respond to their needs (e.g., no time to do outreach, provide culturally relevant services), to keep informed on best practices and new services, and for their own self-care. A few participants from Kelowna also noted that it was sometimes unclear how to best coordinate services across different members due to the lack of role clarity. In Vancouver specifically, participants highlighted that there were not enough staff to meet high workload demands due to unaffordable housing options, which caused providers to move away from areas where services were desperately needed or to hold multiple jobs, further contributing to their burnout. Participants also uniquely described feeling immense anxieties about youths’ lives due to the *“life or death situation”* (Participant 2, small group 3, Vancouver) and the challenge of working within a risk-averse system.

Thus, service providers across the four communities emphasized the need for more support from clinical supervisors/managers and encouragement from their team members for their own mental wellness, as well as less organizational pressures and flexibility in their roles to do what is clinically beneficial.

### System-level innovation

At a systems-level, participants focused primarily on the need for improved navigation of services and increased capacity for local service delivery. These needs intersected with participants’ goals to respond to youths’ needs in the moment and the intra-organizational silos that were encountered. Thus, service providers identified the need for a “*centralized intake point so we can see what services are available, what the wait list looks like, and what are the inclusion, exclusion criteria, so we're not wasting a bunch of time, that's an inefficiency, like huge”* (Participant 1, small group 3, Victoria). Participants also emphasized the need for a provincial clinical records systems to access, with youths’ consent, to improve service continuity.

Additional sub-themes were identified within each community that were distinct to their local context. In Victoria, participants urged for *“more beds, more housing, more outreach workers”* (Participant 1, small group 2, Victoria). This need rested upon the significant waiting lists encountered when making referrals, and youth having to travel outside their communities for services (e.g., to Vancouver) or to local neighborhoods they were trying to avoid.

In Vancouver, participants stressed that the lack of “*coordinated system-level leadership*” to the youth opioid crisis underpinned their organizational and individual-level needs. In these discussions, service providers drew comparisons to other chronic conditions or past crises where there was strong leadership that resulted in significant advancements in prevention, treatment, and research:

*“From a systemic perspective, there's a lack of leadership. For perspective, when the HIV crisis was happening there was like a cause and how to get there and leadership and you have that a little with the opioid crisis but not anything specific to youth. And so, because of that, everybody is just working from such saddled approaches maybe and then I think you need something to channel it a little bit more and get it out there. So, I think there's no leadership in this crisis, especially for youth. And who is going to take that leadership role? I don't know because we're constantly just putting out fires and not responding to the actual crisis itself.”* (Participant 4, small group 1, Vancouver).

Meanwhile in Prince George, service providers described the drug toxicity crisis as being relatively recent in this region of the province, which included remote communities. This led them to prioritize a wider distribution of information about opioid use, services, and MOUD to better reach youth, families/caregivers, and service providers across this large geographic region. In contrast to the other communities, this experience also resulted in their unique emphasis on best practice standards being locally relevant and feasible:

*Participant 1: I'd like to know more just what the best practices look like… cuz there's guidelines and things that have been put out and it all looks good on paper, but it's not necessarily feasible.*

*Participant 3: Or I’ve been doing a lot of research into best practices and a lot of times, they're a couple years old, but that's the most recent thing… Or it's from Ontario, is that even relevant to us? Right, so where are the best practices coming from and do they make sense in that context?…[And] whose job is it to keep us up to date? Like are all of us individually supposed to keep up on our desk when we can or should someone in the province be educating us on what we should be implementing for best practice?* (Small group 3, Prince George).

Finally, in Kelowna, participants’ system-level needs focused on a wider acceptance of harm reduction. This need arose when reflecting on the impact that stigma and abstinence-focused approaches have on youth and how harm reduction education in schools, hospitals, and the wider community could lead to earlier intervention and prevention of opioid use.

### Ideas for improving the delivery of youth opioid use services and treatments

Across communities, a total of 209 individual ideas were brainstormed to address service providers’ prioritized needs. The ideas for each need theme were summarized are shown in Table 2, along with representative examples and the relevant community(ies). Eight solutions themes were identified to address the need to respond to youth in a timely and appropriate manner. All communities identified a need to expand service delivery locations and engage youth in service planning and monitoring, which had the highest number of individual ideas. The remaining ideas were specific to Victoria and/or Vancouver, including programs that incorporate recreational activities, and specific interventions for youth using stimulants.

**Table 2:** Service providers’ ideas for improving youth opioid use services/treatments by overarching needs theme

Ideas themes^a^	Community	Select representative idea(s)^b^
Need theme: respond to youth in a timely and appropriate manner (n = 79)		
1. Expand service locations throughout communities and outreach-based services (n = 13)	All communities	“Multiple hubs throughout the community where kids can access most types of treatment”
2. Engage youth in service planning and monitoring (n = 12)	All communities	“Ask youth what services they would like to see”
3. Programs that create or maintain positive relationships in youths’ lives (n = 11)	Victoria	“Allow friends to go to treatment together”
4. Create programs that incorporate recreational activities for youth (n = 10)	Victoria	“Groups for youth using opioids that will take them to do different activities and help them discover a hobby or passion”
5. Integrate diverse staff and experiences into service delivery (n = 10)	Victoria	“Value lived experience” “Provide accessibility and accommodations for staff”
6. Create programs that develop or maintain cultural connection (n = 9)	Victoria	“Connection to land” “Cross cultural services (not a one size fit)”
7. Create programs and spaces that are specific to youth and diverse youth (n = 7)	Victoria; Vancouver	“Youth specific services whenever possible, e.g., ‘sobering centre’, OAT, stabilization, etc.” “Identity-based groups within services, e.g., queerabilities”
8. Develop interventions for youth using stimulants (n = 7)	Vancouver	“Move towards RCT for stimulant assisted treatment”
Need theme: improve intra-organizational supports and inter-organizational collaboration (n = 66)		
1. Inter-organizational activities (e.g., newsletters, social networking sites, tours) or events (e.g., conferences) to learn about what other agencies/providers offer (n = 39)	All communities	“Quarterly meeting for all youth services in Prince George—a big conference” “Weekly newsletter via email that discusses different services”
2. Activities to build up service provider competencies (n = 17)	All communities	“Gather collective wisdom through community of practice”
3. Procedures that enable inter-organizational collaboration (n = 10)	All communities	“Build reciprocal and positive relationships with other organizations”
Need theme: system-level innovation (n = 64)		
1. Develop infrastructure to increase local service/treatment capacity (n = 30)	Victoria; Vancouver	“Designate a number of units in buildings as affordable youth units” “Fund grassroots community initiatives”
2. Create clearer pathways into and between services/treatments (n = 14)	Victoria; Vancouver	“One information source for treatment information and referrals” “Different levels of treatment in one place, e.g., day program, residential, counseling, groups”
3. Reduce unnecessary system-related barriers to access services/treatments (n = 14)	Prince George; Victoria	“Shared consent forms for continuity of care” “Simple universal referral forms with minimal information required”
4. Develop youth-specific standards (n = 6)	Vancouver	“Develop indicators of success from a social lens and beyond urine drug screens and emergency department visits”

^a^Ideas were brainstormed by individual participants in the workshops and documented on flip charts. The overarching need themes (Fig. 1) were used to sort individual ideas from across all four communities. Individual ideas were then studied and thematically analysed following an inductive approach. Data shown reflect the semantic ideas themes, which represent clusters of individual ideas that were similar across participants and communities. Data in the brackets represent the number of individual ideas that were collated into the idea theme, thus a higher number represents a higher number of individual ideas coded in the respective ideas theme

^b^Data shown are representative examples of individual ideas that were coded within the semantic ideas themes

For the overarching need to improve intra-organizational supports and inter-organizational collaboration, the most frequently referenced ideas focused on activities or events that could promote providers’ knowledge of other resources in their community. Examples included regularly distributed newsletters about different organizations, tours of other agencies, social networking sites for service providers to share information, and local conferences bringing all youth service providers together. Several ideas about how to promote service providers’ competencies were also identified, primarily focused on communities of practice, and other unique ideas including book clubs for staff and shadowing opportunities at other clinics. A smaller number of ideas revolved around improving inter-organizational collaboration, such as integrated case management, interagency partnerships to facilitate group-based services, and developing protocols that outline collaborating organization’s roles.

For the overarching system-level innovation need, there was greater variation across the communities in the patterns of ideas themes. In Victoria and Vancouver, many ideas focused on how the system could develop the infrastructure to increase local service/treatment capacity, such as increasing provincial funding allocation through fundraising or re-allocating taxes towards youth housing and services. Additionally, system navigators, integrated services, and provincial service directories/databases were brainstormed to create clearer service pathways. In Vancouver specifically, different types of youth-specific standards (e.g., indicators of success, inclusion and exclusion criteria for services) were identified as solutions to operationalize youth-specific best practices. Meanwhile, universal referral forms and shared consent forms were identified in Prince George and Victoria to reduce unnecessary red tape when accessing services/treatments.

## Discussion

This study applied human-centered co-design methods to identify opportunities for improving the quality of youth opioid use services and treatments. The multi-site qualitative analysis led to the development of three overarching opportunities that reflected points of connection across the four communities, while also supporting understanding of how local context shaped those themes. Thus, this in-depth analysis provides critical insight into the service providers’ experiences and directions for improving youth opioid use services and treatments within and beyond these local communities.

The first theme described participants’ priorities to deliver services that address youths’ individual needs in the moment. In Vancouver and Kelowna, service providers emphasized youths’ complex needs (e.g., concurrent mental illnesses, housing insecurity, stimulant use), while in Victoria and Prince George concerns were raised about how to best support youth who identify as 2SLGBTQIA + or Indigenous. Across communities, participants spoke to the challenges of providing youth with developmentally appropriate services throughout the care continuum, due to the lack of youth-specific environments or specialized providers in youth substance use. They also emphasized the importance of building and maintaining respectful and non-judgmental relationships with youth and engaging youth in treatment planning and delivery. To better respond to the unique needs of youth, service providers suggested numerous solutions, such as engaging youth in service/treatment planning, providing youth with programs that help them maintain meaningful connections (i.e., positive relationships, cultural connection, and recreational activities), and creating services that are youth-specific. Importantly, these findings from service providers resonate with studies that have been done with youth using opioids and other drugs [13, 18, 21, 23, 24]. Youth in many settings have articulated their challenges accessing environments that are adult-oriented [13, 21], engaging in treatments that apply a ‘one-size fits all’ ideology [23], and that do not reflect their unique goals and preferences [18, 22–24, 37]. This suggests that service providers and youth may have similar priorities during their point-of-care interactions. And yet, this combination of evidence strongly indicates that neither youth nor service providers’ priorities are being met.

The second and third overarching themes support explanations for these priorities being unmet. At an organizational-level, service providers across communities shared the difficulties they had aligning services to youths’ needs due to time constraints and their limited skills/training and relationships with other agencies. These barriers led participants to prioritize intra-organizational supports and inter-organizational collaboration to respond to youth in a more timely and appropriate manner. Relatedly, service providers identified several systems-level challenges that perpetuated these barriers, such as poor coordination and consistency of procedures and policies across sectors; inadequate resources, services, and funding to meet demands; and limited best practice guidelines. To our knowledge, only one other study has addressed service providers’ perspectives on gaps in youth opioid use services/treatments [38]. In this recent survey-based study (n = 154), Nairn et al. identified several organizational and systems-level opportunities that align with those of our qualitative study, including the need for a continuum of care that enables continuity between settings or integrated service environments [38]. Nairn et al. also identified similar accessibility barriers (e.g., wait lists, limited localized service options), limited youth-specific services, and inadequate funding as critical gaps [38]. While not specific to opioid use services/treatments, these challenges have been raised by service providers in another recent study focused on youth substance use services more broadly [39]. Results from our research extends these prior studies by providing solutions to these challenges. For instance, inter-organizational activities (e.g., newsletters, networking visits) may improve continuity and consistency between organizations, while competency building activities (e.g., community of practice) may address the barrier of limited youth-specific services.

Our in-depth qualitative findings elucidate how challenging these gaps were for service providers as they experienced tremendous fears over youths’ safety, burnout, and pressures to uphold their professional goals and responsibilities, which were constantly *“undermined”* by the system’s shortcomings. These findings connect to broader socio-institutional theory [40], which describes the interdependencies among micro-level (patient care), meso-level (healthcare institutions), and macro-level (health care policy) perspectives. Research in other healthcare settings (see for example, [41–44]) have shown that healthcare professionals’ experiences and practices (micro-level perspectives) are shaped by multiple interacting influences at higher organizational and institutional levels (meso-level perspectives). Our findings also reflect a tension that arises when policies at macro-levels are incongruent with micro-level needs [45]. In the context of drug policy, this may stem from the longstanding challenge of abstinence-oriented ideologies that limit the implementation of evidence-based interventions to curb the individual and community-level impacts of drug use [46]. In our study, service providers across all communities reflected on how such policies were compromising and undermining their quality of care at an individual youth-level, but in slightly different ways. For example, participants in Prince George described how MOUD best practices, which are determined by provincial guidelines (i.e., macro-level) and require daily access to MOUD dispensing pharmacies, were not reflective of their community’s needs, particularly for those living in remote communities. The ongoing disconnect between provincial policies and the local needs of communities has been further evident in recent provincial policies for the decriminalization of possessing up to 2.5 g of illicit substances for personal use, an amount which has been criticized as being negligible for those living in rural and remote communities who must travel long distances to acquire illicit substances [47].

These tensions beg the question of how system-level policies can be developed to meet individual youth and community needs, and, thereby, those of community-based service providers. To that end, service providers identified over 200 solutions to their overarching needs. The system-level ideas in particular may resolve some of these multi-level tensions. For example, developing youth-specific indicators of opioid use service quality, that include domains other than abstinence or acute care visits, may increase consistency across providers and agencies while encouraging a more youth-centered, evidence-based, timely, and wider continuum of care. Similarly, developing infrastructure to increase local service capacity may allow communities to take stronger leadership in implementing services that reflect the evolving local needs of youth and enhancing collaboration and resource distribution between local organizations. Additionally, creating provincial communities of practice could bring micro-, meso-, and macro-level perspectives from each region together to inform the development and implementation of provincial policies. This would ensure that service providers and organizational leads from all regions of the province are represented and that the local needs of each community are reflected in policies, including rural and remote communities that are disproportionately affected by the opioid crisis [29, 30].

## Limitations

While our study applied original methods that allowed us to gather in-depth data for improving youth opioid use services, there are important limitations that must be acknowledged. In particular, human-centered co-design is a participatory approach to co-designing products or services that are grounded in the needs of the end-users (i.e., service providers). This approach allowed us to identify opportunities to improve youth opioid use services/treatments that were grounded in the lived experience and expertise of service providers. However, data collection occurred in single community-based workshops and analysis occurred thereafter. This limited our ability to probe on some of the within-site themes, which would have enriched their contextualization.

## Conclusions

This multi-site study provides in-depth evidence to inform opportunities to improve the quality of youth opioid use services and treatments. Importantly, this research focused on service providers who have had limited opportunities to inform this evidence, despite their significant roles and responsibilities in the delivery of these services. Our findings emphasize the need for an integrated multi-level response that includes systems-level innovation, intra-organizational support and inter-organizational collaboration, and individual-level care that is timely and developmentally appropriate for youth.

## Data Availability

The datasets generated and/or analysed during the current study are not publicly available due as participants did not consent to public disclosure of their data.

## Abbreviations

MOUD: Medications for opioid use disorder
CBPR: Community-based participatory research
ITT: Improving treatment together project
BC: British Columbia
